# Synthesis, Surface Engineering, and Emerging Applications of Black Phosphorus Nanosheets

**DOI:** 10.3390/nano16211389

**Published:** 2026-10-29

**Authors:** Rakshya Panta, Howyn Tang, Lina Elrofaie, Jin Zhang

**Affiliations:** 1 School of Biomedical Engineering, University of Western Ontario, London, ON N6A 5B9, Canada; rpanta@uwo.ca (R.P.); htang248@uwo.ca (H.T.); 2 Department of Chemical and Biochemical Engineering, University of Western Ontario, London, ON N6A 5B9, Canada; lelrofai@uwo.ca

**Keywords:** black phosphorus, black phosphorus nanosheets, phosphorene, surface engineering, synthesis, optical properties, nanomedicine, biosensing

## Abstract

Black phosphorus nanosheets (BPNS) are emerging two-dimensional (2D) materials with exceptional optical and electronic properties, making them promising candidates for a wide range of nanotechnology applications. Their distinctive structural, electronic, and optical characteristics, particularly their thickness-dependent direct band gap, which varies from approximately 0.3 eV in bulk black phosphorus to 2.0 eV in the monolayer, have been extensively investigated using angle-resolved photoemission spectroscopy (ARPES) and density functional theory (DFT) calculations. This review focuses on the anisotropic optical responses of BPNS, including their polarization-dependent linear absorption and reflection, as well as nonlinear optical phenomena such as saturable absorption and the Kerr effect. In addition, the potential applications of BPNS in solar cells, sensors, and biomedical devices are discussed, with particular emphasis on their tunable band gap and enhanced sensitivity. Their reported biodegradability and generally favorable biocompatibility in preclinical studies further support their potential in nanomedicine, although these properties depend on formulation, dose, and surface modification. Despite these promising properties, challenges related to large-scale synthesis and environmental stability remain. Addressing these limitations through scalable synthesis approaches and improved stability will be essential for translating the unique capabilities of BPNS into practical applications.

## 1. Introduction

Since the isolation of graphene, two-dimensional (2D) materials have attracted tremendous interest owing to their unique electronic, optical, mechanical, and chemical properties. Black phosphorus nanosheets (BPNS), a layered allotrope of phosphorus, were first isolated through mechanical exfoliation, nearly a decade after the discovery of graphene [1]. Compared to other 2D nanosheets, e.g., graphene, hexagonal boron nitride (hBN), transition metal dichalcogenides (TMDs), and layered double hydroxides (LDHs) [2,3,4], BPNS show unique anisotropic optical properties [5] and good biocompatibility, and has emerged as one of the most intensively investigated 2D materials for its potential in a wide range of biomedical applications such as drug delivery, biosensing, photothermal therapy, and biological imaging, enabling enhanced precision in therapeutic interventions and diagnostic processes [6,7].

Black phosphorus (BP) crystallizes in the orthorhombic A17 phase (*Cmca*), where phosphorus atoms form a puckered sp^3^-like lattice with distinct armchair and zigzag directions. Weak van der Waals interactions between adjacent layers enable exfoliation into BPNS, including monolayer phosphorene (~0.53 nm thick) [3,8]. This unique structure gives rise to pronounced in-plane anisotropy, leading to direction-dependent electronic, optical, thermal, and mechanical properties [5,9]. BPNS exhibit a thickness-dependent direct band gap that can be tuned continuously from 0.3 eV in the bulk of BP crystals to 2.0 eV in the monolayer [10], originating from quantum confinement and reduced interlayer coupling as the number of layers decreases. Compared with graphene, which lacks an intrinsic band gap, BPNS offer a finite and thickness-dependent band gap that is useful for transistor and photodetector applications. However, TMDs also exhibit thickness-dependent electronic structures; for example, molybdenum disulfide (MoS_2_) changes from an indirect-gap semiconductor in the bulk form to a direct-gap semiconductor in the monolayer [11]. The electronic band structure of monolayer MoS_2_ can also be modified by dielectric screening from the surrounding environment [12]. BPNS are therefore distinguished not simply by band gap tunability, but by the combination of a thickness-dependent direct band gap, relatively high carrier mobility, and pronounced in-plane anisotropy, which is particularly useful for infrared and polarization-sensitive optoelectronic devices [3,5,10]. In addition, field-effect transistors (FETs) based on few-layer BPNS exhibited room-temperature mobilities of up to 300 cm^2^ V^−1^ s^−1^ and drain current modulation exceeding 10^3^, while low-temperature measurements revealed on/off ratios greater than 10^5^ [13]. Furthermore, the relatively high Seebeck coefficient and anisotropic in-plane Young’s modulus, ranging from approximately 44 to over 90 GPa depending on crystallographic orientation, suggest significant advantages for thermoelectric and mechanically robust device applications [14].

Beyond their exceptional electronic and optoelectronic properties, BPNS exhibit significant advantages in biomedicine. Phosphorus is an essential biological element, and BP-based nanomaterials can undergo oxidative degradation to phosphate- and phosphonate-containing products under aqueous and physiological conditions. However, their biological response depends strongly on particle size, dose, surface chemistry, coating, and the surrounding degradation environment. In vitro studies have generally reported acceptable cell compatibility within the tested concentration ranges, whereas selected in vivo studies using surface-modified BP formulations have demonstrated biodegradation and clearance with limited short-term toxicity [15]. These findings are promising for biomedical applications, but they should not be interpreted as evidence of universal biocompatibility or long-term safety. Surface functionalization can substantially alter degradation kinetics, cellular uptake, circulation, and immune interactions, and further systematic in vivo studies are required to establish the long-term biosafety of BPNS-based systems [15,16].

This review focuses on recent progress in the synthesis, surface modification, and emerging applications of BPNS. Both top-down and bottom-up synthesis methods are discussed, followed by surface modification strategies. The review then examines the key optical, electronic, thermal, mechanical, and magnetic properties of BPNS arising from their anisotropic crystal structure and bonding characteristics. Emphasis is placed on surface engineering, which plays a critical role in improving environmental stability and tuning nonlinear optical responses as well as biomedical functionality. By linking atomic structure with physicochemical behavior, this work establishes a clear relationship between surface modification and functional performance in optical, electronic, and biomedical systems. Overall, the review provides an integrated framework for understanding BPNS as multifunctional materials and highlights current challenges and future directions for their scalable and practical applications.

## 2. Synthesis of BPNS

The synthesis route plays a critical role in determining the morphology, crystallinity, defect density, oxidation stability, lateral dimensions, and electronic performance of BPNS. Historically, bulk BP was first obtained by subjecting white or red phosphorus to high pressures (>1 GPa) at elevated temperatures, driving conversion to the layered A17 phase upon decompression [17].

Modern synthesis strategies can be broadly classified into three categories: (i) top-down exfoliation methods, which isolate phosphorene from bulk BP crystals; (ii) bottom-up growth methods, which construct BPNS from molecular or vapor-phase phosphorus precursors; and (iii) phase-conversion approaches, where red or white phosphorus is transformed into the orthorhombic BP phase under high pressure. Each route produces BP nanostructures with distinct physicochemical properties that determine their suitability for applications in nanoelectronics, optoelectronics, catalysis, sensing, and biomedicine [18,19]. 

### 2.1. Top-Down Exfoliation Approaches

Top-down methods start from layered bulk BP crystals and delaminate them into thinner structures by mechanical force, liquid-phase energy input, or electrochemically driven intercalation. These techniques differ in achievable lateral size, thickness control, yield, and defect/oxidation levels.

#### 2.1.1. Mechanical Exfoliation

Analogous to the “Scotch-tape” method that launched graphene research, micromechanical exfoliation repeatedly cleaves bulk BP crystals with adhesive tape or visco-elastic PDMS stamps. The process is carried out entirely inside an argon or nitrogen glovebox to protect freshly exposed surfaces from oxidation. Thin platelets are transferred onto 285 nm silicon dioxide (SiO_2_)/silicon (Si) wafers, where optical contrast permits rapid layer-count estimation, followed by atomic force microscopy (AFM) and Raman spectroscopy for precise thickness assignment. Although the yield of isolated flakes is limited (10^−2^% of the starting crystal), mechanically exfoliated flakes retain pristine lattice order, negligible basal-plane defects, and carrier mobility approaching that of the parent bulk crystal. Early few-layer BPNS FETs displayed on/off current modulation exceeding 10^5^ and room-temperature hole mobilities near 1000 cm^2^ V^−1^ s^−1^ when contacted with high-work-function metals [1]. Four-probe measurements on one- and two-layer phosphorene further confirmed its intrinsic semiconducting nature, ambipolarity, and gate-tunable band gap [13].

#### 2.1.2. Liquid-Phase Exfoliation (LPE)

To increase the production throughput, bulk BP crystals are ground under inert gas, dispersed in a high-boiling, polar aprotic solvent whose surface energy matches that of BP before exfoliation (e.g., N-methyl-2-pyrrolidone, N-cyclohexyl-2-pyrrolidone, dimethylformamide), sealed in a glovebox vial, and sonicated for 6–12 h. In particular, dimethyl sulfoxide (DMSO) is an effective solvent for producing high-quality phosphorene flakes through liquid-phase exfoliation (LPE), although the water content must be carefully controlled to optimize nanosheet quality and stability [20]. Cavitation micro-jets and shear forces overcome interlayer attractions, producing colloidal nanosheets. Sequential centrifugation fractions the dispersion by thickness and lateral size, and AFM-calibrated optical extinction coefficients enable rapid dimensional analysis. Electronic-grade dispersions can reach concentrations of 0.1–0.4 mg mL^−1^, while vacuum-filtered films or ink-jet-printed lines yield ambipolar thin-film transistors with on/off ratios up to 10^4^ and mobilities of 10–50 cm^2^ V^−1^ s^−1^ after mild annealing to remove solvent residues [21,22]. Adsorbed solvent molecules retard, but do not eliminate, oxidation, and surfactant-assisted aqueous LPE and noncovalent amphiphile wrapping further extend processing to biologically relevant media while providing steric protection [23].

Solution routes that subject phosphorus precursors (bulk BP powders, red phosphorus slurries, or organophosphorus compounds) to elevated temperature and autogenous pressure in sealed vessels can yield ultrasmall BPNS following topochemical conversion and/or exfoliative cutting. For example, ultrasmall (<5 nm) black phosphorous quantum dots (BPQDs) have been prepared by dispersing bulk BP in polar solvents followed by high-energy treatment (probe and bath sonication) and size-selective purification, producing photothermally active, biodegradable nanomaterials for imaging and therapy [24]. One-pot solventless or polymer-assisted adaptations further improve colloidal stability and enable surface functionalization [25]. The extreme quantum confinement in BPQDs widens the effective band gap toward the visible, enhances photoluminescence (PL), and accelerates oxidative degradation features that can be exploited in bioimaging but require passivation for electronic uses [24].

#### 2.1.3. Electrochemical Intercalation/Exfoliation

Electrochemical methods accelerate delamination by driving ion insertion and lattice expansion. A bulk BP crystal serves as the working electrode in an aprotic electrolyte containing tetrabutyl-ammonium (TBA^+^). Applying a potential below 3 V intercalates the TBA^+^ between layers, thereby weakening van der Waals cohesion. A brief low-energy sonication then releases 2–6-layer nanosheets with large (few-micrometer) lateral dimensions and alkyl-terminated edges [26]. Integrating intercalation with vacuum filtration, Jeon et al. produced centimeter-scale, uniform BPNS networks that functioned as near-infrared (NIR) phototransistor arrays with excellent pixel-to-pixel repeatability [27]. Electrochemical control also tunes defect density, making this route attractive for catalysis and energy-storage electrodes.

#### 2.1.4. Ball Milling

High-energy ball milling is another top-down approach used to produce BPNS from bulk BP. During milling, repeated mechanical impact and shear forces reduce the lateral dimensions and thickness of the layered material and promote exfoliation into nanoscale sheets. Compared with micromechanical exfoliation, ball milling is more suitable for larger-scale processing, although the high mechanical energy can introduce structural defects, reduce lateral dimensions, and increase the susceptibility of freshly generated surfaces to oxidation. Processing under an inert atmosphere is therefore generally required to limit degradation. Ball milling has also been combined with surface functionalization, allowing exfoliation and chemical modification to occur simultaneously. However, milling media can introduce impurities. Recent reviews have identified high-energy ball milling as a relevant route for the scalable preparation of BPNS [28].

### 2.2. Bottom-Up and Reconstructive Growth Approaches

Bottom-up methods seek to synthesize BPNS directly from molecular, vapor, or solution precursors, often through reconstructive transformations of other phosphorus allotropes, or vapor/mineralizer-mediated assembly on catalytic substrates. While still less mature than top-down exfoliation for producing device-grade flakes, these processes are essential for scale-up, film uniformity, and integrated manufacturing.

#### 2.2.1. Chemical Vapor Transport/Mineralizer-Mediated Growth

The tin/tin(IV) iodide (Sn/SnI_4_) transport reaction is widely used for bulk BP platelet growth. Most mechanical or chemical exfoliation experiments begin with centimeter-scale BP crystals grown in sealed silica ampoules by a tin-iodide “short transport” reaction. Red phosphorus is combined with tin (Sn) and tin(IV) iodide (SnI_4_), flame-sealed, and heated so that SnI_4_ vapor carries phosphorus as tin-phosphorus iodide species from a 650 °C hot zone to a 550 °C cool zone, where BP crystals form through mineralizer-assisted growth and gas-phase transport [17,29]. The reaction operates at the tube’s autogenous pressure and avoids the multi-GPa pressures required for Bridgman growth, while still producing high-quality BP platelets with three characteristic Raman vibrational modes at 358–362, 432–439, and 460–467 cm^−1^ and narrow $A_{g}^{1}$ Raman linewidths (full width at half maximum (FWHM) of ~2 cm^−1^) [30,31].

Variants that include gold (Au)/Sn alloy solvents, alternative halide mineralizers, or modified thermal gradients have been explored to enlarge crystal domains and reduce nucleation density [29,32]. The underlying mechanism involves the formation of volatile tin and phosphorus iodides at the hot end of a sealed ampoule, followed by their transport and disproportionation in a cooler zone, where bulk BPNS platelets crystallize. Careful selection of ampoule geometry, temperature ramp rates, and mineralizer ratios enables control over platelet thickness and reduces inclusion of metallic intergrowths; however, Sn and iodine (I) residues, as well as Au-containing ternaries in older protocols, can persist and influence electronic and electrochemical behavior [29]. This sealed-ampoule process yields bulk BP crystals several millimeters across for subsequent exfoliation, rather than directly producing few-layer films [32].

#### 2.2.2. Vapor-Phase Deposition/Chemical Vapor Deposition Growth

True epitaxial chemical vapor deposition of crystalline BP films is challenging because of phosphorus’s high vapor pressure, reactivity, and multiple allotropes. Nonetheless, vapor-phase strategies that deliver a controlled flux of tetraphosphorus (P_4_; or other P_x_ species) to catalytic surfaces under tailored temperature/pressure gradients have produced sub-millimeter BPNS domains and oriented films. Process windows are narrow: insufficient phosphorus overpressure favors amorphous deposits; excessive phosphorus flux or temperature excursions promote red phosphorus formation or sublimation. Progress in substrate engineering, precursor metering, and growth kinetics modeling continues to push lateral dimensions upward and thicknesses downward toward device-relevant regimes. Comprehensive analyses of growth thermodynamics and kinetic barriers across the phosphorus-allotrope BPNS-landscape guide future scale-up [3]. In one seed-assisted approach, gold-tin-polyphosphate (Au_3_SnP_7_) nuclei supported the growth of BP films with lateral dimensions in millimeters and thicknesses of a few to hundreds of nanometres [19].

### 2.3. Phase Conversion and High-Pressure Synthesis Method

#### 2.3.1. High-Pressure Conversion Approaches

Historically, bulk BP was first obtained by subjecting white or red phosphorus to high pressures (>1 GPa) at elevated temperatures, driving conversion to the layered A17 phase upon decompression [17]. Modern high-pressure synthesis uses multianvil or belt presses that enable controlled pressure–temperature (P-T) paths because crystallite size, mosaicity, and defect density depend sensitively on pressure hold time, maximum temperature, and cooling rate. Depending on processing conditions, high-pressure-converted BP may contain structural defects, residual precursor phases, or contaminants introduced by the pressure medium or capsule materials [28,33]. In contrast, Sn/SnI_4_-assisted short-distance vapor transport can produce millimeter-scale BP crystals without multi-GPa pressure [32].

#### 2.3.2. Pulsed Laser Deposition (PLD) Followed by High-Pressure/Temperature Conversion

A recently demonstrated scalable route decouples deposition from crystallization. Amorphous red phosphorus films are first deposited by pulsed laser ablation in an inert argon atmosphere. The deposited amorphous phosphorus film is subsequently converted into crystalline BP through high-pressure (~8 Pa) and moderate-temperature treatment (∼150–350 °C) in diamond-anvil or belt-type high-pressure apparatuses. Post-conversion annealing sharpens Raman features and improves crystallinity. Continuous centimeter-scale films ~18 nm thick and exhibiting mid-infrared PL near the bulk BP band edge (~0.3 eV) have been reported, demonstrating centimeter-scale conversion and highlighting a potential pathway to wafer-scale integration [34]. Another approach involved depositing red phosphorus onto flexible polyester before high-pressure conversion at room temperature, resulting in nanocrystalline BP films 40 nm thick and 4 mm across [27].

### 2.4. Comparison of Major Synthesis Routes

The selection of an appropriate BPNS synthesis strategy depends strongly on the intended application. Mechanical exfoliation is preferred for fundamental electronic studies because it produces BPNS with exceptional crystallinity, minimal structural defects, and high carrier mobility [35]. Liquid-phase and electrochemical exfoliation are more suitable for scalable production of BPNS for biosensing, flexible electronics, and photothermal therapy, although they differ in yield, lateral size, and defect density [22,36,37,38]. Hydrothermal synthesis is ideal for producing BPQDs for imaging and nanomedicine due to strong quantum confinement, enhanced PL, and biodegradability [39,40]. Vapor-phase deposition methods show considerable promise for wafer-scale fabrication of BP films for photonic and optoelectronic devices, while high-pressure synthesis remains indispensable for producing high-quality bulk BP crystals and for investigating the phase stability of BP under extreme conditions [33].

Overall, each synthesis strategy offers distinct advantages and limitations in terms of crystal quality, scalability, defect density, thickness control, and environmental stability. Consequently, the synthesis method directly influences the structural, optical, electronic, and biomedical properties of BPNS discussed in the following sections. The demonstrated thicknesses, lateral dimensions, yield, production scale, defects/impurities, and oxidation stability are compared in Table 1. It should be noted, however, that quantitative yields and oxidation lifetimes are not consistently reported across methods, and can only indicate route-specific observations and qualitative limitations.

## 3. Surface Modification of BPNS

BPNS exhibit excellent electronic and optical properties; however, their practical applications are fundamentally limited by rapid degradation under ambient conditions. The lone-pair electrons on surface phosphorus atoms react spontaneously with oxygen, water, and light, leading to oxidation into phosphorus oxides and eventual structural collapse of the lattice. This degradation can occur within hours in unprotected BPNS, severely diminishing carrier mobility, PL, and photothermal efficiency. Therefore, surface modification is not an optional step but a critical requirement that determines whether BPNS can transition from laboratory synthesis to real-world applications. Effective stabilization strategies must simultaneously suppress oxidation, preserve intrinsic anisotropy, and allow functional integration for biomedical, electronic, and photonic systems.

### 3.1. Covalent Functionalization Strategies

#### 3.1.1. Aryl Diazonium Chemistry

Aryl diazonium chemistry has been extensively used to form covalent bonds on the surfaces of nanomaterials such as graphene and carbon nanotubes [42,43,44]. In this process, the diazonium salts capture an electron from the lone pair of surface phosphorous atoms, creating a highly reactive phenyl radical and releasing nitrogen molecules. This results in the formation of a phosphorus–carbon (P-C) covalent bond on the surface of BPNS [45]. These modified BPNS showed significant stability in morphology even when exposed to environmental conditions [45]. Moreover, FETs fabricated with these modified BPNS demonstrated a notable improvement in both the on/off current ratio and carrier mobility compared to those utilizing untreated BPNS.

**Figure 1:**
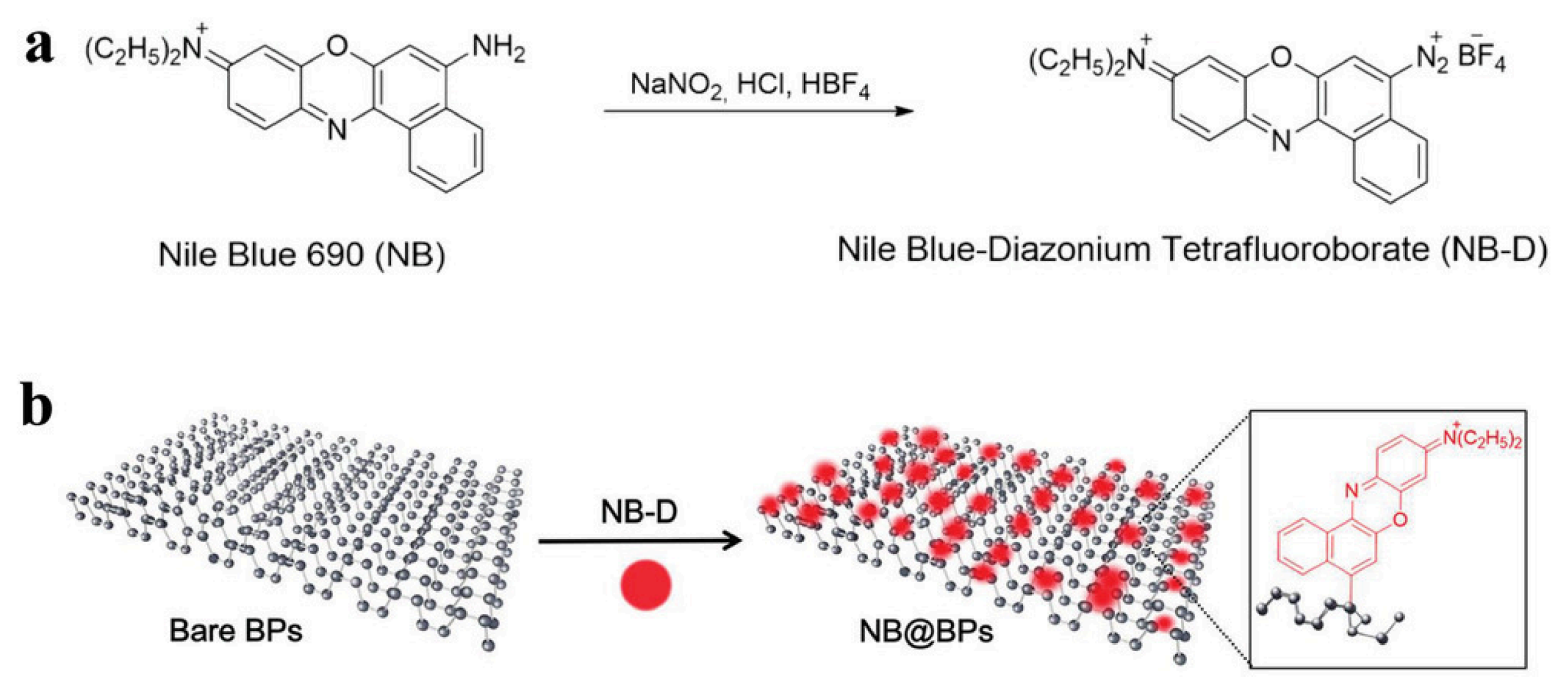
Schematic diagram of (**a**) the production process of NB-D and (**b**) Schematic illustration of NB-D (red circles) functionalization of BPNS. Reproduced with permission from Zhao et al., Chemistry of Materials; published by ACS Publications, 2017 [46].

Zhao et al. utilized this technique with BPNS-based nanomaterials and examined their potential in the medical field. A diazonium fluorescent dye, Nile Blue 690 (NB), was converted into its diazonium tetrafluoroborate salt (NB-D) and then covalently conjugated to the surface of BPNS through a P-C bond to create NB@BPNS, which could emit heat and fluorescence when exposed to NIR light as shown in Figure 1 [46]. Following 3 days of exposure to air and water, the absorbance of NB@BPNS decreased by only 3%, compared to approximately 40% for untreated BPNS [46]. Consequently, while untreated BPNS only raised the solution temperature to 18.4 °C under NIR irradiation, NB@BPNS increased it to 25.3 °C, suggesting that the photothermal activity of BPNS was effectively maintained by surface modification. Subsequently, the NB@BPNS solution was introduced to mice with tumors through tail vein injection and observed a continuous red fluorescence signal in the tumors and some organs after 1 day, disappearing completely after 48 h, indicating the clearance of BPNS from the body through the reticuloendothelial system. As anticipated, tumors treated with NB@BPNS experienced a temperature rise of 23.5 °C, sufficient for tumor ablation, while those treated with untreated BPNS only raised by 11.5 °C due to BPNS degradation in the bloodstream. No significant direct effects were observed in any group where the treatment took place. These results underscore the promising potential of NB@BPNS in NIR fluorescence imaging and photothermal therapy.

#### 3.1.2. Metal-Ion Coordination Chemistry

Hua et al. introduced organic semiconductors (OSCs)-coated polyacrylonitrile (PAN) nanofibrous mats as responsive chemo-sensors for 2,4,6-trinitrotoluene (TNT) and hydrogen peroxide (H_2_O_2_) vapor. Incorporating conjugated polymers like poly(triphenylamine-alt-biphenylene vinylene) (TPA-PBPV) and small organic molecules onto electrospun nanofibers resulted in a significant 9-fold rise in fluorescence intensity and a 14-fold boost in sensitivity [47]. The enhancement results in a high area-to-volume ratio and evanescent-wave effect. The substitution of organic semiconductors broadens the scope of detection applications. These OSC-coated PAN nanofibrous mats demonstrated superior sensitivity for TNT and H_2_O_2_ compared to pristine OSC films, with sensitivity inversely proportional to nanofiber diameter.

Yu et al. used titanium amino-benzenesulfonate complexes (Ti-SA4) to enhance antibacterial activity in BPNS-based nanomaterials, achieving ratios of 99 ± 1% against *E. coli* and 95 ± 2% against *Staphylococcus aureus* compared to 40 ± 4% and 34 ± 6% with BPNS alone [48]. Ti-SA4@BPNS rapidly inactivated bacteria within 3 h at 50 μg mL^−1^ due to strong phosphorus-titanium (P-Ti) coordination, enriching SA4 on BPNS surfaces. Platinum (Pt) coordinated with BPNS using modified cisplatin-Pt-nitrate (NO_3_^−^), maintaining smooth surfaces and photothermal activity for over 24 h. Pt@BPNS improved cellular uptake, sensitizing cisplatin-resistant cancer cell lines A2780 and HepG2 to cisplatin [49]. BPNS carried 1,2-diaminocyclohexane platinum(II) (DACHPt) at twice their weight through coordination, producing BPNS/DACHPt with enhanced stability and photothermal performance, leading to complete tumor growth inhibition after a two-week treatment [50]. Trimethyl dichlorosilane (TMSCl) stabilized BPNS, maintaining morphology and photothermal properties for 24 h with no cytotoxicity at 200 ppm. TMSCl@BPNS inhibited HeLa and HepG2 cell growth by 60% after 808 nm laser irradiation for 24 h, surpassing the inhibition rate of bare BPNS (approximately 40%) [51].

#### 3.1.3. Hydrophobic and Covalent Polymer Interaction

In biomedicine, coating BPNS or quantum dots with polymer chains turns them into polymer-based nanoparticles that protect the reactive phosphorus surface, lower surface energy, neutralize charge, and prevent agglomeration, thereby boosting biocompatibility and sharply reducing oxidative degradation [52]. Because polymers can be engineered with specific ligands, pH triggers, or photolabile bonds, they enable “smart” BPNS systems that home to tumors, release drugs on command, or report their location by fluorescence. The remainder of this subsection surveys the main polymer strategies applied to BPNS-based nanomaterials and itemizes their demonstrated uses in cancer theranostics, tissue engineering, bioimaging, wound healing, and biosensing [15].

#### 3.1.4. Electrostatic Adsorption of Cationic Polymers

Liquid-exfoliated BPNS have a negative zeta potential (−15 to −40 mV) and adsorb cationic polymers like amine-functionalized polyethylene glycol (PEG-NH_2_), polyethyleneimine (PEI), and polydopamine (PDA) through electrostatic interaction. Polyethylene glycol (PEG)-ylation decreases aggregation, maintaining stability in phosphate-buffered saline (PBS) and cell medium for a week without affecting ultraviolet–visible–near-infrared (UV-vis-NIR) absorbance or photothermal activity (conversion efficiency approximately 29.8%) [23]. PEGylated BPNS can load up to 108% doxorubicin (DOX) or cyanine7 (Cy7) for cancer chemotherapy or in vivo NIR imaging, respectively. Conjugating PEG with targeting molecules like folic acid enhances cellular uptake for targeted cancer treatment [24]. PEI maintains photothermal stability and conversion efficiency, allowing gene–drug delivery. Black phosphorus-polyethyleneimine (BP-PEI) nanosheets deliver small interfering RNA (siRNA) for surviving knockdown, achieving synergistic anti-tumor effects. Multifunctional polymers like chitosan-polyethylene glycol-folic acid (CS-PEG-FA) and polyethylene glycol-polyethyleneimine (PEG-PEI) create combined therapy systems with targeted drug release and bioimaging [53].

#### 3.1.5. Edge-Selective Modification Strategy

**Figure 2:**
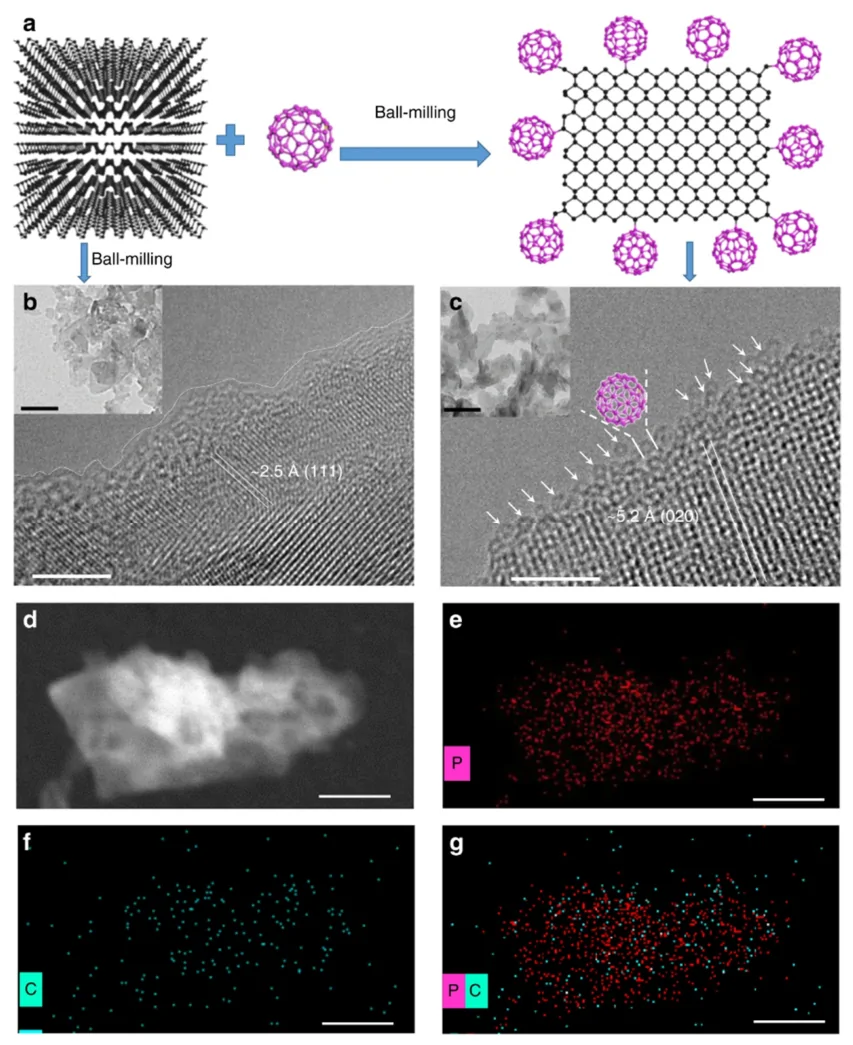
Covalent functionalization of black phosphorus nanosheets (BPNS) via high-energy ball milling. (**a**) Schematic illustration of the ball-milling process used to covalently graft fullerene (C_60_) molecules onto BPNS. (**b**) High-resolution TEM image of pristine BPNS before functionalization (scale bar = 5 nm). (**c**) High-resolution TEM image of the functionalized BPNS, showing the attachment of C_60_ molecules (arrows) at the nanosheet edges (scale bar = 5 nm). (**d**–**g**) scanning TEM and energy dispersive X-ray spectroscopy carbon (C) and phosphorus (P) mapping images of BPNS-C_60_ (scale bar = 100 nm). Reproduced with permission from Zhu et al., Nature Communications; published by Nature, 2018 [54].

Zhu et al. developed an edge-selective modification strategy to stabilize BPNS using buckminsterfullerene (C_60_) molecules. As shown in Figure 2, bulk BP and C_60_ were mixed at a 1:2 mass ratio and subjected to mechanochemical ball milling [54]. The mechanical energy simultaneously exfoliated BP into few-layer nanosheets and generated highly reactive phosphorus sites at the nanosheet edges which formed covalent P-C bonds with C_60_ molecules. Unbound C_60_ was removed by Soxhlet extraction with carbon disulfide (CS_2_). The resulting BP-C_60_ hybrid effectively protected the chemically vulnerable BP edges from oxidation and degradation, while minimizing modification of the basal plane, thereby preserving the intrinsic properties of BP.

#### 3.1.6. Hydrophobic Interaction

Silk fibroin (SF), originating from silk, demonstrates high biocompatibility and versatility in biomedical contexts. Huang et al. applied SF to coat BPNS, forming stable BPNS@SF layers through hydrophobic interactions [55]. Unlike BPNS@N-methyl-2-pyrrolidone (NMP) solutions, which degraded rapidly, BPNS@SF solutions remained stable for up to 14 days. While initial absorbance levels were similar, BPNS@NMP absorbance decreased over time, contrasting with the unchanged absorbance of BPNS@SF. BPNS@SF maintained photothermal effects and exhibited minimal cytotoxicity and hemolysis. SF’s processability facilitated the fabrication of various BPNS-based materials with sustained photothermal activity. BPNS@SF wound dressings effectively eliminate bacteria under NIR light and promote wound healing [56].More recently, Alunni Cardinali et al. investigated black phosphorus/silk fibroin films against *Candida albicans* and showed that incorporation of BP altered the silk fibroin structure and increased film stiffness, which suppressed the filamentous and invasive growth of *C. albicans*. The films also reduced fungal cell vitality and biofilm-forming capability, further supporting the potential of BP/SF composites as bioselective coatings for biomedical applications [57].

### 3.2. Cytomembrane Encapsulation

Bio-inspired methods such as ligand joining, and cell membrane encapsulation are gaining popularity. Hu et al. pioneered cell membrane coating for nanoparticles, offering anti-phagocytic properties and prolonging their lifespan in vivo [58]. Various cell membranes, including those from red blood cells (RBCs), leukocytes, stem cells, and cancer cells, are utilized to encapsulate nanoparticles for diverse applications. RBC membrane coating protects from immune surveillance, while leukocyte membrane coating extends blood circulation [59]. Stem cell membranes improve targeted drug delivery, and cancer cell membranes facilitate tumor recognition.

**Figure 3:**
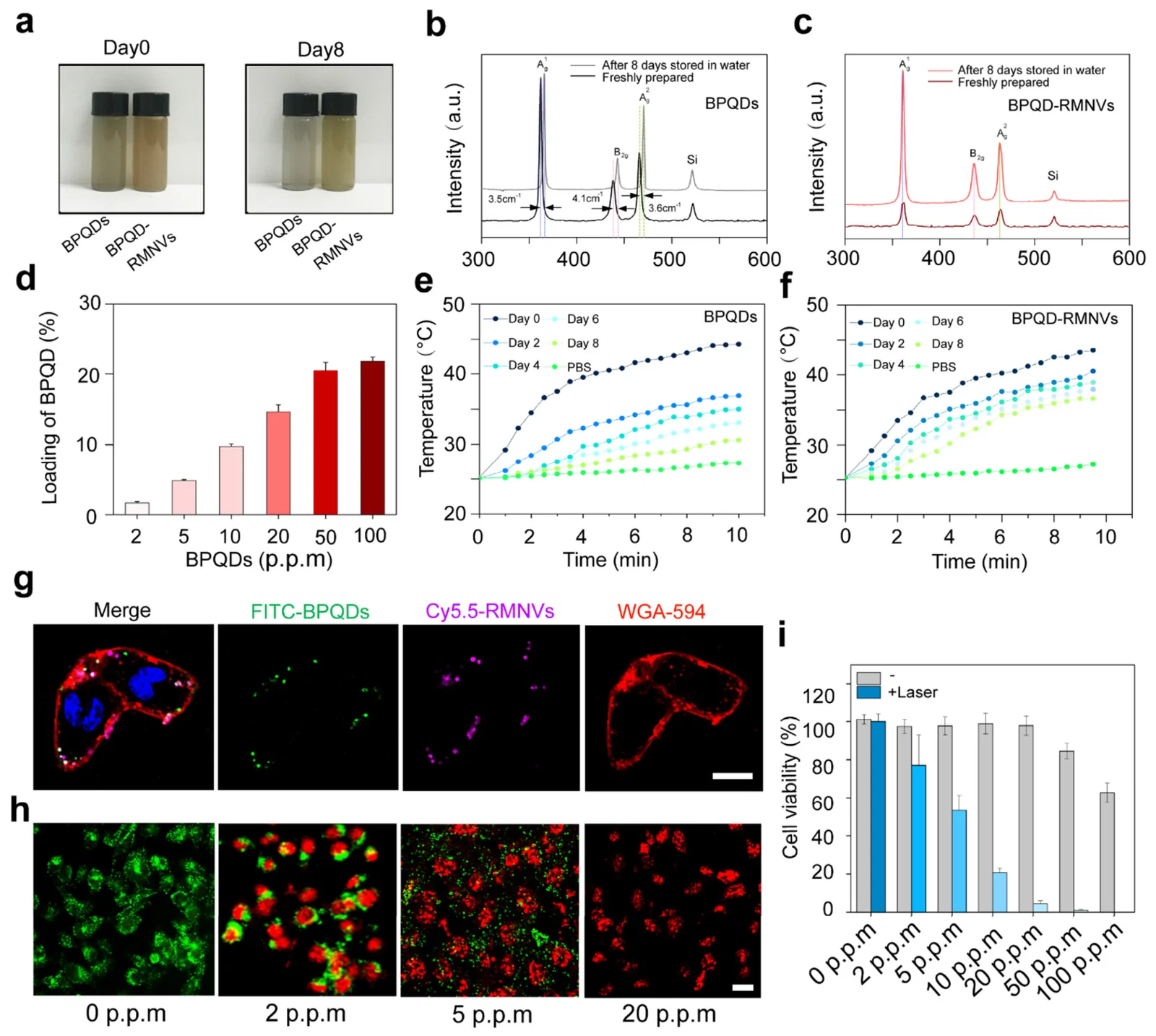
Characterization and biological evaluation of erythrocyte membrane-coated black phosphorus quantum dot nanovesicles (BPQD-RMNVs). (**a**) Photographs showing the appearance of BPQDs and BPQD-RMNVs after storage in water for 0 and 8 days. (**b**,**c**) Raman spectra comparing the structural stability of BPQDs and BPQD-RMNVs following storage. (**d**) Encapsulation efficiency of BPQDs within red blood cell membrane nanovesicles at different BPQD concentrations. (**e**,**f**) Temperature profiles demonstrating the photothermal performance of BPQDs and BPQD-RMNVs after storage for different time periods under laser irradiation. (**g**,**h**) Fluorescence microscopy images illustrating the cellular uptake and intracellular localization of membrane-coated BPQDs. (**i**) Cell viability of treated cells with and without laser exposure, demonstrating the photothermal therapeutic effect of BPQD-RMNVs. Reproduced with permission from Liang et al., Journal of Controlled Release; published by Elsevier, 2019 [60].

RBC membranes, known for their non-immunogenicity, are commonly employed for producing long-circulating nanoparticles. Liang et al. encapsulated negatively charged BPQDs with RBC membranes, creating core–shell nanovesicles (NVs) that shield BPQDs from degradation. BPQD-RMNVs maintained their photothermal properties and showed prolonged circulation and tumor accumulation in vivo. This underscores the significant enhancement of BPQDs’ systemic circulation and targeting efficiency achieved through RBC membrane coating. As shown in Figure 3, erythrocyte membrane coating improves the stability and functional performance of BPQDs: the coated BPQD-RMNVs retain their structural integrity and photothermal response during storage and exhibit effective cellular uptake and laser-induced cytotoxicity. Together, these results demonstrate that membrane encapsulation can protect BPQDs from degradation while preserving their photothermal therapeutic function [60].

### 3.3. Other Strategies

Previous studies have shown that organic solvents like n-cyclohexyl-2-pyrrolidone (CHP) and NMP can create a protective layer on BPNS, thereby improving stability, but they need thorough washing due to toxicity [61]. Sun et al. utilized DMPU, a less toxic solvent, to raise BPNS yield and minimize oxidation, leading to superior biocompatibility [62]. Hu et al. innovated by combining various modification methods to produce BPNS@copper (Cu), enhancing photothermal efficacy and enabling combined hemodynamic and photothermal therapy [63]. Cancer cell membranes (CCMs) were utilized for tailored cancer vaccines, showcasing enhanced photothermal performance and boosting the activity of tumor-specific CD8+ T cells [64].

## 4. Optical Properties of BPNS

The optical response of BPNS originates directly from their unique puckered orthorhombic crystal structure. The distorted honeycomb lattice creates two distinct in-plane crystallographic directions, armchair and zigzag, which differ significantly in bond angle distribution, orbital overlap, and charge delocalization. Consequently, BPNS exhibit pronounced in-plane anisotropy, including direction-dependent effective masses, dielectric responses, and optical transition dipole moments. Light polarized along the armchair direction couples more efficiently with electronic states than light polarized along the zigzag direction, giving rise to pronounced linear dichroism and birefringence [14,65]. In addition to in-plane anisotropy, interlayer van der Waals coupling results in the characteristic band gap of BPNS [66]. This structural evolution enables broadband optical tunability across the visible to mid-infrared region. Therefore, the optical properties of BPNS are governed by a dual structural mechanism: in-plane anisotropic bonding and out-of-plane interlayer electronic coupling. These structural features not only determine the fundamental optical absorption characteristics of BPNS and emission behavior but also directly govern vibrational responses, polarization selectivity, excitonic transitions, and nonlinear light–matter interactions. Consequently, nearly all optical phenomena observed in BPNS can be traced back to the coupling between its anisotropic lattice geometry and thickness-dependent electronic band structure [67,68].

### 4.1. Raman Scattering

Because Raman-active phonon modes arise from atomic vibrations, their frequencies and intensities are highly sensitive to symmetry of BPNS crystal, layer number, and bonding environment. The orthorhombic lattice gives rise to distinct vibrational modes associated with both intralayer phosphorus–phosphorus (P-P) bond stretching and interlayer van der Waals interactions, making Raman spectroscopy an effective tool for probing BPNS structure [68,69]. Since BPNS are a layered material, the typical Raman spectrum for BPNS has both high-frequency intralayer Raman modes and low-frequency interlayer Raman modes as well as their vibrational motions [65]. Depending on the thickness (number of layers) of BPNS, and the number of atoms in the unit cell, different numbers of high-frequency and low-frequency Raman modes can be observed [14]. Under Raman scattering spectroscopy, the principal first-order Raman-active modes of BP occur near 360, 440, and 470 cm^−1^ and are commonly assigned to the $A_{g}^{1}$, $B_{2g}$, and $A_{g}^{2}$ modes, respectively [65,66]. Due to the anisotropic nature of BPNS, the intensities of the Raman modes can change and show symmetry and periodicity when the polarization of incident or scattered light is changed [65]. For example, when polarized incident light travels along the *a*-axis, the Raman mode at 470 cm^−1^ has twice the intensity compared to when the polarized incident light travels along the *b*-axis [66]. This angular dependence originates from direction-dependent Raman tensor elements caused by unequal atomic displacement responses along the armchair and zigzag axes. As a result, polarized Raman spectroscopy can be used not only to identify BPNS thickness but also to determine crystal orientation [70,71].

### 4.2. Thickness-Dependent Optical Properties

The thickness-dependent band gap of BP bridges the gap between zero-band gap graphene and many semiconducting TMDs, which typically possess band gaps in the approximately 1–2 eV range [14,65]. The reported band gap for bulk BP to monolayer is 0.3 eV to 2.0 eV [1,72,73]. As BPNS thickness increases, the band gap decreases. The band gap is approximately 0.3 eV for bulk BPNS, 0.8 eV for triple-layered BPNS, 1.3 eV for double-layered BPNS, and 2.0 eV for the monolayer [10]. It should also be noted that the band gap in BPNS is temperature-dependent. Unlike many conventional semiconductors, whose band gaps decrease with increasing temperature, BPNS can exhibit a positive temperature coefficient over the reported temperature range [74].

**Figure 4:**
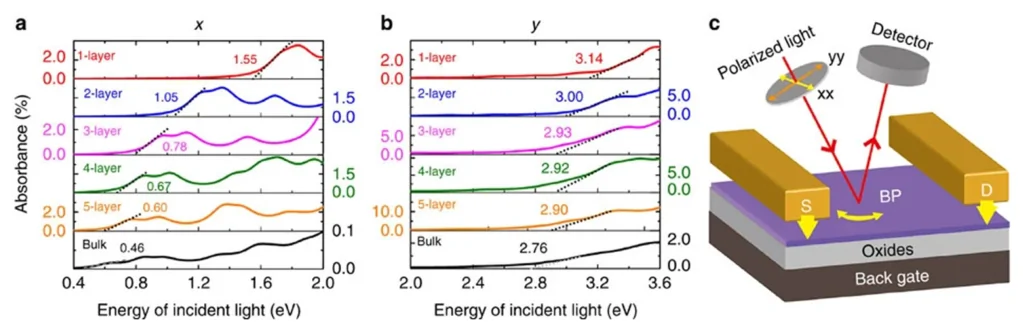
Thickness- and polarization-dependent optical absorption of few-layer black phosphorus (BP). The black dotted lines represent approximate linear fits used to determine the band edges of the first absorption peak, which are anisotropic between the x and y directions. (**a**) Optical absorption spectra for light polarized along the armchair (x) direction, showing a decrease in the absorption edge from approximately 1.55 eV for monolayer BP to 0.60 eV for five-layer BP. (**b**) Optical absorption spectra for light polarized along the zigzag (y) direction, where the absorption edge changes only slightly from approximately 3.14 to 2.90 eV. (**c**) Schematic illustration of the polarized optical measurement configuration proposed for determining the crystallographic orientation of few-layer BP. Reproduced with permission from Qiao et al., Nature Communications; published by Nature, 2014 [5].

The absorbed light is polarized in the armchair (x) direction in the polarization window of 1.1 eV to 2.8 eV but exhibits strongly suppressed absorption for light polarized along the zigzag direction within the specified energy range [75]. Depending on the thickness of BPNS, the low end of the polarization window can be adjusted from 1100 to 300 meV, which covers the infrared and NIR frequencies. BPNS also exhibit strong linear dichroism. As illustrated in Figure 4, this optical anisotropy is strongly dependent on both BPNS thickness and crystallographic direction. For light polarized along the armchair (x) direction, the absorption edge decreases substantially from approximately 1.55 eV for monolayer BP to 0.60 eV for five-layer BP as the number of layers increases. In contrast, for light polarized along the zigzag (y) direction, the absorption edge changes only slightly, from approximately 3.14 eV to 2.90 eV from monolayer to five-layered BPNS.

**Figure 5:**
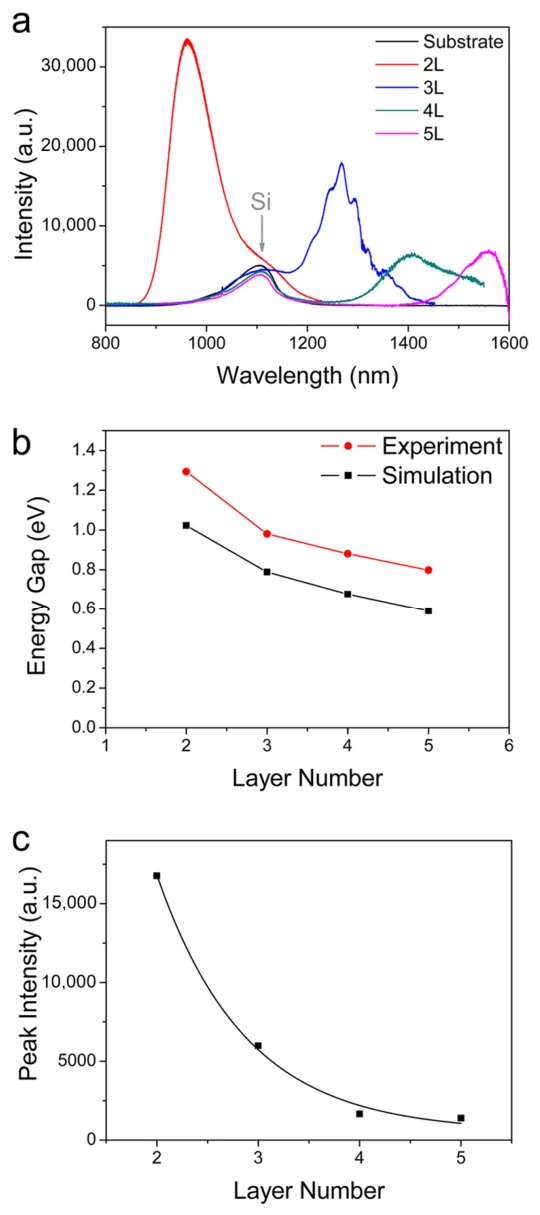
Layer-dependent photoluminescence and energy gap of few-layer black phosphorus. (**a**) Photoluminescence spectra of the substrate and bilayer (2L), trilayer (3L), tetralayer (4L), and pentalayer (5L) BP, showing a progressive shift in the PL peak toward longer wavelengths with increasing layer number. (**b**) Experimental and simulated energy gaps as a function of layer number, showing a decrease in energy gap with increasing BP thickness. (**c**) Dependence of PL peak intensity on layer number, demonstrating a pronounced reduction in emission intensity with increasing thickness. Reproduced with permission from Zhang et al., ACS Nano; published by ACS Publications, 2014 [77].

Similarly, the PL of BPNS is also strongly polarization-dependent. As the light polarization angle θ changes, the PL intensity has a cos^2^θ pattern, consistent with the lack of PL under incident light polarized in the zigzag (y) direction [76]. Furthermore, the PL emission wavelength (λ_em_) of BPNS also depends on thickness. As the number of layers increases, the λ_em_ is longer with lower photon energies (Figure 5). In addition to the spectral shift, the PL peak intensity decreases substantially with increasing BP thickness, as shown in Figure 5c. Together, these results demonstrate the strong layer dependence of the optical properties of few-layer BP, with increasing thickness resulting in a lower transition energy and weaker PL emission [77].

Furthermore, Zhang et al. performed a comprehensive infrared characterization of BPNS with thicknesses ranging from two to fifteen layers. It was found that few-layer BPNS have a unique infrared spectrum that is thickness-dependent and has a series of absorption resonances that can serve as an infrared fingerprint [78].

### 4.3. Nonlinear Optical Properties

The nonlinear optical properties of a material refer to those light–matter interactions where more than one photon interacts with the atoms/molecules of the material [79]. High-intensity light, such as that of a laser, is usually required to observe nonlinear optical properties. Under these conditions, enough atoms/molecules can be excited and produce vibrations of different frequencies, generating new frequency waves that mix to create nonlinear optical effects. In comparison, linear optics occur in low-intensity light, and the number of interactions where atoms/molecules interact with more than one photon/wave is negligible. This review covers two of the nonlinear optical properties of BPNS: the saturable absorption effect (SA) and the Kerr effect. BPNS exhibit particularly strong nonlinear optical behavior because their direct band gap, anisotropic carrier dynamics, and high optical absorption enable efficient interaction with intense electromagnetic fields. Moreover, their thickness-dependent electronic structure allows nonlinear responses to be tuned through structural engineering [80,81].

#### 4.3.1. Saturable Absorption Effect (SA)

SA is a phenomenon where the transmitted light’s intensity increases as the incident light’s intensity increases [79]. The SA of BPNS is enhanced by its direct band gap and broadband optical absorption, which permit efficient excitation of charge carriers over a wide spectral range. The anisotropic electronic band structure also contributes to direction-dependent absorption saturation behavior [82,83]. It is caused by Pauli blocking effects. Under intense excitation, available electronic states near the band edges become occupied, reducing the probability of additional interband transitions through Pauli blocking and thereby decreasing the absorption [84,85]. The SA of BPNS follows the following equation:

(1) $$\frac{dI}{dz}=-\alpha \left(I\right)I$$

where *I* is the optical intensity, α(*I*) is the intensity-dependent absorption coefficient, and z is the propagation coordinate [86]. BPNS SA has been verified by multiple studies using the Z-scan measurement technique on BPNS dispersions [22,86,87]. Hanlon et al. found that few-layer BPNS dispersions had increased transmission under femtosecond pulses at 1030 and 515 nm [22]. Another study explored BPNS nanoplatelets, which exhibited SA under femtosecond excitation at 400 nm and 800 nm [87]. Picosecond pulses at 1562 nm and 1930 nm also showed SA. The one-photon absorption model can be used to describe the mechanisms of BPNS SA, as seen in the following equation:

(2) T=1−αs1+IIs−αns

where *T* is the transmittance, *I* is the input intensity, *I_s_* is the saturation intensity at which the saturable component of the absorption decreases to one-half of its low-intensity value; *α*_s_ is the modulation depth, and *α_ns_* is the nonsaturable loss [86,87].

Although graphene and several TMD-based saturable absorbers have been widely studied, their modulation depth, spectral coverage, or long-wavelength performance may be limited for particular laser systems. BP exhibits broadband saturable absorption extending from the NIR toward the mid-infrared, making it attractive for ultrafast photonic applications [88]. As such, there is a lack of saturable absorbers around 3 µm. However, BPNS possess broadband SA in the wavelength range of 1 µm to 2.7 µm, which demonstrates BPNS as a potential saturable absorber at longer wavelengths around 3 µm [88]. The effect of laser pulse duration on BPNS SA has also been investigated. The saturable intensity (*I_s_*) has an inverse relationship with pulse duration, while the nonlinear absorption coefficient and figure of merit have a direct relationship with pulse duration [86].

#### 4.3.2. Kerr Effect

The optical Kerr effect is a third-order nonlinear optical phenomenon in which the refractive index of a material varies with the intensity of the incident optical field. The strong Kerr nonlinearity observed in BPNS is closely associated with their anisotropic electronic structure and large third-order optical susceptibility. The anisotropic puckered lattice produces direction-dependent electronic polarizability, which contributes to pronounced nonlinear refractive-index modulation under intense optical excitation [89,90]. For an applied optical electric field *E*, the induced polarization *P* can be expanded as a power series of the field:

(3) $$P=\varepsilon_{0}\left(\chi^{\left(1\right)}E+\chi^{\left(2\right)}EE+\chi^{\left(3\right)}EEE+\ldots \right)$$

where *P* is the induced polarization, *E* is the electric field amplitude, *ε*_0_ is the vacuum permittivity, and *χ*^(*n*)^ is the n-order susceptibility [10,91]. *χ*^(1)^ is the susceptibility, which describes the linear optical response of the material, *χ*^(2)^ describes two-field interaction effects, and *χ*^(3)^ describes three-field interaction effects. The real part of *χ*^(3)^ is the Kerr effect [91]. The Kerr effect is most directly described by

(4) $$n\left(I\right)=n_{0}+n_{2}\;I$$

where *n*_0_ is a linear refractive index, *n*_2_ is a nonlinear refractive-index coefficient, and *I* is optical intensity. For an isotropic, non-magnetic, and weakly absorbing medium with the assumption of a linearly polarized optical field, a small nonlinear refractive-index change, and the conventional degenerate third-order approximation, the nonlinear refractive-index coefficient is related to the real part of the third-order susceptibility by

(5) $$n_{2}=\frac{3Re\chi^{\left(3\right)}}{4n_{0}^{2}\varepsilon^{0}c}$$

where *n*_0_ is the linear refractive index and *c* is the speed of light in vacuum. This scalar relationship represents an isotropic approximation, and does not fully correspond to the optical response of anisotropic BP. For anisotropic BP, the third-order susceptibility is a fourth-rank tensor, and the nonlinear refractive-index coefficient depends on tensor components and interactions with the applied optical field. Therefore, the measured Kerr response depends on the crystallographic direction, polarization configuration, wavelength, pulse duration, sample thickness, and experimental configuration [91]. The nonlinear refractive index of BPNS nanoparticles was measured by Z-scan measurement technique and found to be approximately (6.8 ± 0.2) × 10^−13^ m^2^ W^−1^ [92]. In another study, the nonlinear refractive index of BPNS is estimated to be approximately 10^−5^ cm^2^ W^−1^ and the third-order nonlinear susceptibility (*χ*^(3)^) is approximately 10^−8^ esu [93]. It is important to note that the reported values may differ under different experimental conditions and are not universal constants.

## 5. Electronic Properties and Charge Transport of BPNS

### 5.1. Electronic Properties

**Figure 6:**
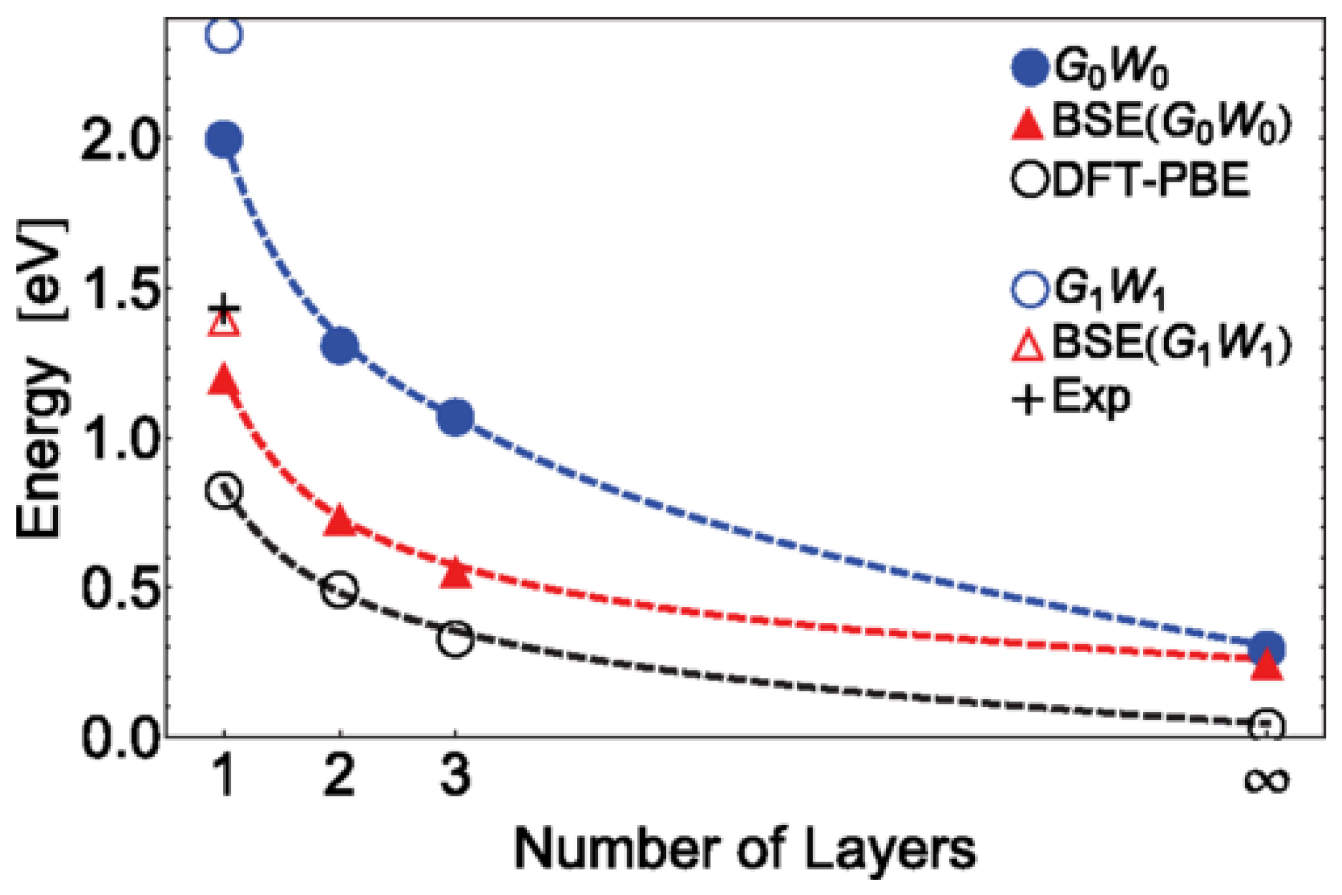
Layer-dependent evolution of the electronic band gap in black phosphorus from the bulk material to monolayer phosphorene. Reproduced with permission from Tran et al., Physical Review B; published by APS Journals, 2014 [75].

BPNS possesses a direct band gap at all thicknesses, a rare feature among 2D materials. As mentioned in Section 4.2., BPNS has a band gap of 0.3–2.0 eV depending on its thickness which bridges the band gap range between graphene and TMDs. Hence, BPNS can function across a wide spectral range, from the visible to the mid-infrared. This makes it particularly attractive for optoelectronic applications, including broadband photodetectors, light-emitting diodes (LEDs), and photovoltaic cells, where precise energy band alignment is essential for optimal device performance [3,85,94]. As illustrated in Figure 6, the electronic band gap of BP is strongly dependent on the number of layers, increasing progressively as the material is thinned from bulk BP to monolayer phosphorene [75].

The crystal structure of BPNS results in differing band dispersion, dielectric constants, and transition of dipole moments along the two axes, giving rise to a direction-dependent response to electric fields and optical excitation. This anisotropic behavior influences conductivity, absorption, and recombination rates, making BPNS suitable for devices that benefit from directional sensitivity, such as polarization-dependent detectors and anisotropic conductors [42,94]. BPNS-based FETs exhibit ambipolar transport, meaning both electron (n-type) and hole (p-type) conduction can be modulated through gate voltage. This behavior is highly beneficial for logic device design, particularly in complementary metal-oxide-semiconductor (CMOS)-like circuits, where both n- and p-type devices are needed. The ability to tune the carrier type through electrostatic gating allows for greater flexibility in electronic architecture and enables dynamic adjustment of device characteristics in response to external stimuli [3,13].

### 5.2. Electron Transport Properties of BPNS

**Figure 7:**
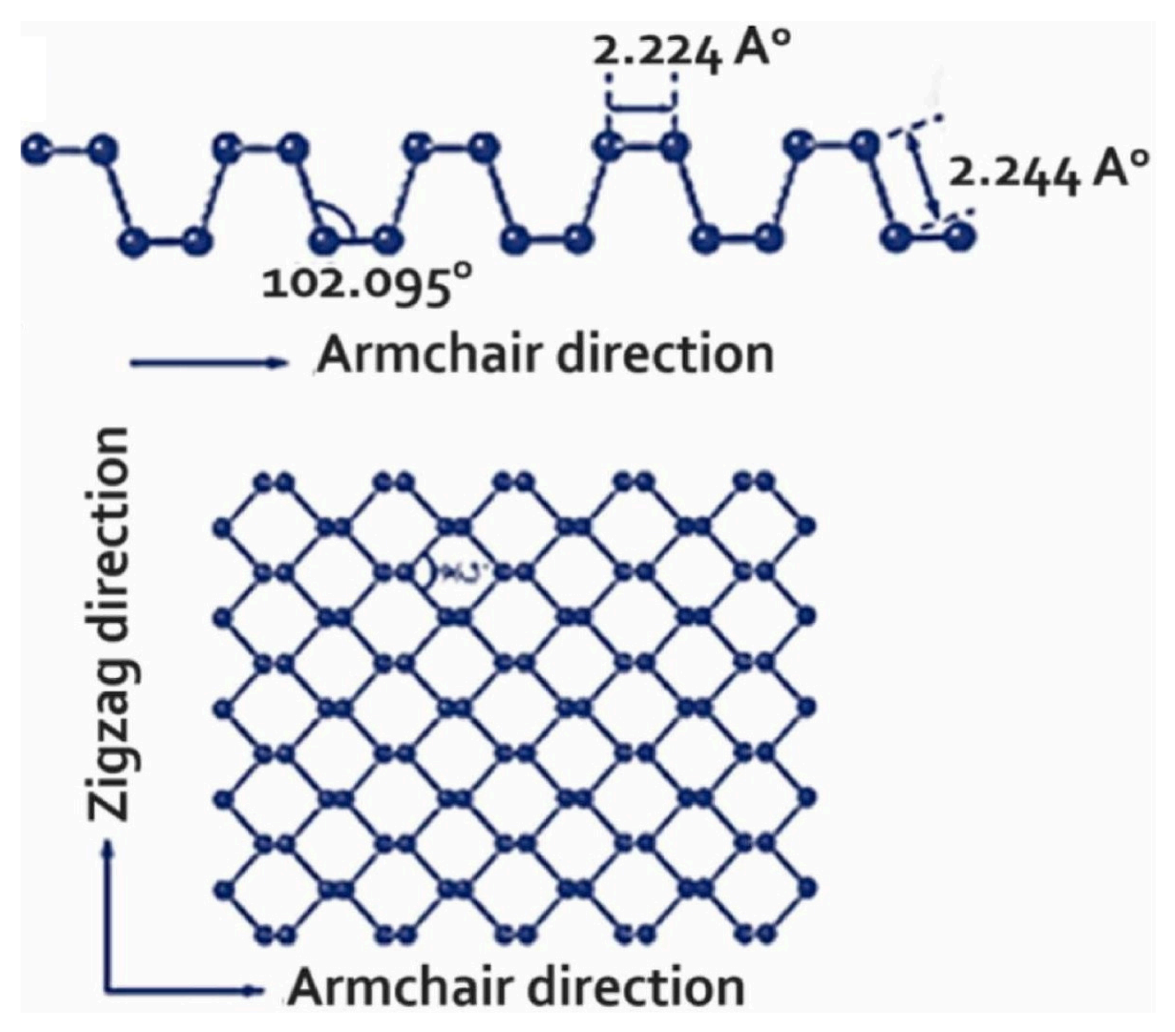
Puckered crystal structure of black phosphorus showing the in-plane armchair and zigzag crystallographic directions. The structural anisotropy gives rise to direction-dependent charge transport and other physical properties. Reproduced with permission from Mishra et al., Next Materials; published by Elsevier, 2024 [8].

One of the most advantageous properties of BPNS is its high hole mobility. Experimentally measured hole mobility in few-layer BP devices can approach ~1000 cm^2^ V^−1^ s^−1^ under optimized conditions, although the reported value depends strongly on layer thickness, crystallographic orientation, substrate, device fabrication, and environmental protection. This high mobility is due to the low effective mass of holes in this direction and relatively weak phonon scattering in high-quality samples. However, mobility decreases with increasing thickness due to interlayer interactions and increases in effective mass. Additionally, environmental exposure degrades mobility over time unless the material is encapsulated. Despite this, the carrier mobility of BPNS remains superior to many other 2D semiconductors, offering the potential for high-speed, low-power electronics [3,94,95]. Furthermore, BPNS’s direction-dependent electronic properties are caused by charge carriers experiencing different effective masses and transport characteristics along the two crystallographic axes [8]. Charge carriers, both electrons and holes, move more efficiently along the armchair axis than the zigzag axis, where the effective mass is significantly higher (Figure 7). The directional nature of charge transport also influences thermal conductivity and Joule heating behavior, which are critical in device reliability and energy efficiency [94,95,96].

In alignment with its anisotropic crystal structure, BPNS demonstrate direction-dependent charge transport (armchair or zigzag; Figure 7). BP offers a compelling platform for emerging spintronic technologies due to its combination of minimal spin–orbit coupling, long spin coherence times, and anisotropic spin transport. Spin lifetimes in BPNS have been observed to exceed one nanosecond, with directional variations up to sixfold between different crystallographic axes. This makes BPNS an attractive candidate for spin-based memory elements and spin logic devices where low energy loss and directional control are critical. Moreover, its electrically tunable spin transport behavior and gate-modulated spin lifetimes suggest potential for reconfigurable device architectures. Although spintronics remains an early-stage application area for BPNS, its foundational properties demonstrate strong promise, warranting further exploration [97]. A more comprehensive analysis of BPNS’s magnetic and spin-related properties can be found in Section 7.

### 5.3. BP-Based Van Der Waals Heterostructures and Device Behavior

Black phosphorus is commonly dominated by p-type transport under ambient or typical device conditions, although its carrier concentration and transport polarity can be strongly modulated by electrostatic gating. This gate dependence makes BP particularly useful in van der Waals heterostructures, where the relative carrier densities and band alignment of the constituent two-dimensional materials can be tuned electrically. In BP/MoS_2_ heterostructures, for example, gate-controlled modulation of the BP and MoS_2_ channels produce antiambipolar transport and gate-tunable photoresponse over visible and infrared wavelengths. The type-II band alignment also facilitates separation of photoexcited carriers, enabling high photodetector responsivity and detectivity [98]. More recently, an h-BN/MoTe_2_/BP van der Waals heterostructure was used to construct a W-shaped antiambipolar transistor with multiple conductance states. The device showed an on/off current ratio above 10^5^ and could operate as both a multivalued logic element and a gate-tunable photovoltaic photodetector. Importantly, the photocurrent polarity could be reversibly controlled by gate voltage, illustrating how the carrier transport characteristics of BP can be exploited within heterostructures to produce reconfigurable electronic and optoelectronic behavior [99].

### 5.4. Emerging Application of BPNS by Leveraging Its Special Properties

The higher carrier mobility of BPNS, ambipolar behavior, and tunable band gap make it an exceptional material for FETs. These devices exhibit high on/off ratios, low subthreshold swings, and excellent current modulation. The directional dependence of mobility can be harnessed to align device channels for optimal performance, while gate-tunable ambipolarity enables reconfigurable circuit designs. As such, BPNS-based FET hold promises for future integrated circuits operating at reduced power consumption and enhanced speed [3,100]. The direct and tunable band gap of BPNS enables efficient light absorption and emission across a wide spectral range. This makes it suitable for photodetectors sensitive to visible and infrared light, light-emitting devices, and even tunable lasers. Its anisotropic absorption properties allow for the creation of polarization-sensitive detectors, expanding the functionality of BPNS in optoelectronic systems beyond what is possible with isotropic materials [76,77].

The mechanical flexibility of BPNS, combined with its outstanding electronic properties, makes it a strong candidate for use in flexible and wearable electronics. Its ability to maintain high performance under bending and stretching, when properly encapsulated, opens the door to applications in biomedical monitoring, foldable displays, and conformal sensors. Continued improvements in synthesis and encapsulation will be essential for transitioning BPNS from the laboratory to commercial flexible devices [49,53].

## 6. Thermal and Mechanical Properties

### 6.1. Thermal Properties

BPNS exhibit highly anisotropic thermal conductivity due to its puckered, orthorhombic structure. In the in-plane direction, thermal conductivity is much higher along the armchair direction compared to the zigzag direction. This behavior is attributed to the variation in phonon group velocities and anharmonic scattering rates along different crystallographic axes [101]. Experimental studies on few-layer BP have reported strongly anisotropic room-temperature thermal conductivity, with the measured values depending on sample thickness, crystallographic direction, substrate, and measurement method [101,102]. This anisotropy is a direct consequence of BPNS’s unique lattice structure, which restricts heat flow along the puckered axis. Phonons, quantized lattice vibrations, are the primary carriers of heat in BPNS. The strong anharmonicity and low phonon group velocity in certain directions contribute to enhanced phonon scattering, thereby reducing thermal conductivity [103]. Moreover, the presence of intrinsic defects, oxidation, and edge roughness in exfoliated BPNS can introduce additional phonon scattering centers, further suppressing thermal transport. These effects are particularly important for nanoscale devices where heat dissipation is critical.

BPNS begin to oxidize in air even at room temperature, but thermal degradation becomes pronounced at elevated temperatures. Studies have shown that thermal stability is limited to approximately 400 °C under inert conditions, beyond which BPNS transform into other phosphorus allotropes or oxidized derivatives [104]. Under ambient conditions, oxidation-induced degradation accelerates significantly, making thermal protection and encapsulation essential for high-temperature applications.

### 6.2. Mechanical Properties

BPNS exhibit moderate mechanical strength and elasticity relative to other 2D materials. The mechanical properties of BP have been investigated using both theoretical calculations and experimental measurements. These studies show strongly anisotropic elastic behavior, with the reported Young’s modulus depending on crystallographic direction, layer thickness, and measurement method [105,106]. This directional dependence arises from its puckered structure, which allows more deformation along one axis than the other. The breaking strength of monolayer BPNS is estimated to be ~4–5 GPa, lower than graphene but sufficient for flexible device applications. The fracture behavior of BPNS is highly dependent on both its thickness and crystalline orientation. Along the armchair direction, BPNS demonstrate greater flexibility and stretchability due to the more open, accordion-like structure of its lattice [107]. In contrast, the zigzag direction tends to be more brittle. First-principles calculations and experimental tensile tests confirm that BPNS tend to fail along the weaker zigzag direction under mechanical strain. The puckered layers can also accommodate out-of-plane deformation, giving rise to negative Poisson’s ratio effects in some cases.

Compared to other 2D materials, BPNS show promising flexibility, especially in few-layer forms. AFM studies have demonstrated that BPNS can be bent to a significant extent without cracking, especially when encapsulated or supported on flexible substrates [105]. This mechanical flexibility, combined with its high carrier mobility, positions BPNS as an ideal candidate for use in wearable and foldable electronics, where both mechanical compliance and electronic performance are required.

### 6.3. Applications Leveraging Thermal and Mechanical Properties

The anisotropic thermal transport of BP also makes interfacial heat transfer an important consideration in BP-based electronic devices. Experimental measurements have shown strongly orientation-dependent thermal boundary resistance at BP interfaces, indicating that crystal orientation and interfacial structure can significantly influence heat dissipation. These findings suggest potential opportunities for thermal-management applications, although practical use will require improved environmental stability and careful interface engineering [108]. BPNS’s unique combination of mechanical flexibility and electronic/thermal performance makes it suitable for use in flexible electronics. Devices such as bendable sensors, wearable health monitors, and foldable displays can benefit from BPNS’s ability to sustain strain while maintaining structural integrity and heat dissipation capacity [105]. The high elasticity and directional mechanical behavior of BPNS also make it attractive for nanoelectromechanical and microelectromechanical systems (NEMS/MEMS). These devices rely on precise control of mechanical vibrations, stiffness, and resonance frequencies, all of which can be tailored in BPNS via layer number and crystallographic orientation [106].

The anisotropic mechanical properties of BPNS allow for the creation of sensitive strain and pressure sensors. Due to its directionally dependent response to mechanical stress, BPNS can be engineered into strain gauges that can detect subtle deformations, useful in robotics, medical diagnostics, and structural health monitoring [107]. The anisotropic thermal and mechanical responses of BPNS also provide opportunities for thermally managed flexible electronics and mechanically responsive devices. Direction-dependent thermal transport could be exploited in nanoscale devices where controlled heat spreading or thermal isolation is required, while the mechanical flexibility of few-layer BP makes it attractive for flexible transistors, strain-responsive sensors, resonators, and nanoelectromechanical systems. In these applications, crystallographic orientation, layer thickness, substrate support, and encapsulation are important design parameters because they directly influence heat transport, stiffness, deformation, and device stability [101,102,105,106].

## 7. Magnetic and Spin-Related Properties

### 7.1. Anisotropic Spin Transport and Spin Lifetime Modulation

BPNS’s puckered orthorhombic lattice structure leads to direction-dependent behavior of spin-polarized carriers. Specifically, the spin relaxation and spin diffusion processes are more efficient along the armchair direction compared to the zigzag direction. This anisotropy arises from the differences in band dispersion and effective mass along each crystallographic direction. Anisotropic spin transport has been experimentally demonstrated in high-mobility, hBN-encapsulated ultrathin BP spin valves. Cording et al. reported gate-tunable in-plane spin lifetimes exceeding 1 ns and a pronounced dependence of spin transport on spin orientation and crystallographic direction. These experimental observations establish ultrathin BP as a promising platform for studying anisotropic and electrically tunable spin transport, while practical spin-logic and memory applications remain prospective. One of the most promising features of BPNS is its electrostatic gate-tunability. The interplay between field effect and structural anisotropy may further enrich the control possibilities in multilayer or heterostructure configurations [97].

### 7.2. Magnetic Properties: Doping and External Field Effects

Monolayer and few-layer BPNS exhibit diamagnetic behavior under standard conditions due to the absence of unpaired d or f electrons. The weak spin–orbit coupling and lack of intrinsic magnetic ordering render pristine BPNS unsuitable for direct magnetic applications. However, this neutrality provides a clean starting point for controlled induction of magnetism via doping or defect introduction [109]. The magnetic landscape of BPNS can be dramatically altered through transition-metal (TM) doping. Dopants such as vanadium (V), chromium (Cr), manganese (Mn), iron (Fe), and nickel (Ni) introduce localized magnetic moments into the lattice, breaking time-reversal symmetry and potentially enabling ferromagnetic or antiferromagnetic order. First-principles studies suggest that the magnetic behavior depends on dopant position, whether substitutional or interstitial, and on the electronic hybridization with the BPNS host matrix. Pristine phosphorene is non-magnetic; however, first-principles calculations indicate that transition-metal doping can introduce localized magnetic moments and substantially modify its electronic structure. Hashmi and Hong predicted spin-polarized semiconducting behavior for Ti-, Cr-, and Mn-doped phosphorene and half-metallic behavior for V- and Fe-doped systems. These results are theoretical predictions, and experimental validation is still required before applications such as magnetic memory or spin injection can be considered established [109]. Even in the absence of doping, external magnetic fields can influence spin orientation and charge-carrier dynamics. In doped or defective BPNS, magnetic fields further enable control over spin polarization, domain alignment, and magneto-optical responses. This tunability supports applications in magneto-electronic devices, magnetic sensors, and catalysis, where spin-dependent chemical reactions can be modulated by the applied field [109].

### 7.3. Quantum and Interfacial Phenomena

Quantum Hall transport has been experimentally observed in high-quality few-layer BP devices under low-temperature and high-magnetic-field conditions. Encapsulated BP heterostructures exhibit Shubnikov-de Haas oscillations, Landau-level quantization, and integer quantum Hall states, demonstrating the suitability of high-mobility BP for investigating two-dimensional quantum transport. Subsequent studies have also reported fractional quantum Hall features in ultrahigh-quality few-layer BP transistors. These observations establish BP as an experimentally accessible platform for studying anisotropic quantum transport and carrier interactions [110,111]. BPNS’s spin susceptibility, particularly the Pauli spin susceptibility, is significantly enhanced over its bare band susceptibility. This discrepancy arises from strong exchange interactions between charge carriers, indicating the importance of many-body effects. These interactions could play a vital role in collective magnetic phenomena, such as spin density waves or excitonic insulators, especially under doping or gating conditions [104]. Experimental studies have demonstrated room-temperature NH_3_ sensing using exfoliated BP, while theoretical studies have predicted strong and selective interactions of phosphorene with gases such as NO_2_ and NH_3_ [112,113]. These interactions alter the electronic properties of BP and provide a basis for chemiresistive gas sensing. However, direct experimental evidence linking gas adsorption to spin transport or magneto-gas sensing in BP remains limited; therefore, such applications should currently be regarded as prospective rather than experimentally established.

### 7.4. Potential Applications of Magnetic and Spin-Related Properties

The experimentally demonstrated anisotropic and gate-dependent spin transport of ultrathin BP suggests potential opportunities for future spintronic devices [97]. Its long-spin lifetime and strong orientation dependence may be useful for electrically tunable spin transport and directional spin manipulation. However, applications such as spin logic, non-volatile magnetic memory, and spin-based information processing remain largely prospective and require further device-level validation. Similarly, although quantum Hall transport has been experimentally demonstrated in high-mobility BP devices, its translation into practical quantum technologies has not yet been established. Theoretical studies of transition-metal-doped phosphorene further suggest routes for inducing magnetic functionality, but experimental confirmation of these predicted magnetic states remains necessary [109,110].

## 8. Applications of BPNS

BPNS are the most thermodynamically stable allotrope of phosphorus under ambient conditions and have emerged as an important, layered semiconductor for electronics, photonics, sensing, energy storage, biomedicine, and catalysis [114,115,116]. This section covers the applications of BPNS in solar cells, sensors and detectors, nanomedicine, and catalysis. In particular, few-layer BP has emerged as a promising material for heterogeneous photocatalysis because of its broad light absorption, thickness-dependent band gap, and favorable charge-carrier transport properties. BP-based photocatalytic systems have been investigated for processes including water splitting, carbon dioxide (CO_2_) reduction, nitrogen gas (N_2_) fixation, pollutant degradation, and related solar-driven reactions [116]. Recent work has further demonstrated the use of BPNS as a phosphide source for mixed cobalt phosphide clusters active in photocatalytic hydrogen evolution, highlighting the continuing development of BP-based catalytic systems [117].

### 8.1. BPNS Used in Solar Cells

Solar cells, also known as photovoltaic (PV) cells, are devices that convert light into electricity based on the photoelectric effect [118]. In simple terms, when photons from sunlight contact a PV cell, the electrons in the semiconductor materials are excited and freed from the material to move between the layers of the cell and create an electric current. PV systems serve as a clean, renewable, safe, and cheap alternative energy source and are used to power buildings, cars, and supplement power grids. However, the current silicon cells have poor sunlight-to-electrical energy efficiency and are expensive. Hence, research has turned to new PV systems based on organic compounds, quantum dots, excitonic semiconducting materials, dye sensitizers, and perovskite crystals [118]. Its thickness-dependent band gap, excellent carrier mobility, and ambipolar transport property make BPNS an ideal material for solar cell research. Theoretical studies have predicted that phosphorene-based photovoltaic architectures could achieve power conversion efficiencies approaching 20%, depending on the heterostructure configuration, band alignment, and computational assumptions [118,119].

While 2D materials such as TMDs have been used in high-performance thin-film solar cells, their power conversion efficiency (PCE) is not as high as traditional materials such as silicon and cadmium-based materials, largely due to their low carrier mobility [120]. As a result, thin-film solar cell design often incorporates stacking two different types of monolayers to complement their advantageous characteristics. For example, FETs made of monolayer TMD MoS_2_ have a carrier mobility of approximately 200 cm^2^ V^−1^ s^−1^ to 500 cm^2^ V^−1^ s^−1^, while those made of 10 nm thick BPNS have a carrier mobility as high as approximately 1000 cm^2^ V^−1^ s^−1^. Based on density functional theory calculations, Dai and Zeng predicted PCEs of approximately 16% and 18% for MoS_2_ combined with AB- and AA-stacked bilayer phosphorene, respectively. These values are theoretical predictions rather than experimentally measured device efficiencies [121]. A BP/MoSe_2_ van der Waals heterostructure was theoretically proposed to achieve a PCE of up to 23.04%, based on calculated electronic structure, optical absorption, and interfacial charge-separation characteristics [120]. BPNS have also been investigated in inorganic–organic hybrid heterojunction solar cells, which are often easier to fabricate and have decent performance compared to organic heterojunction cells. BPNS was used in conjunction with [6,6]-phenyl-C61-butyric acid methyl ester (PCBM) to form solar cells and had a PCE of 6.4 to 8% [43]. The PCE depended on whether BPNS were doped onto PCBM sheets or doped onto PCBM nanowires before forming knitted sheets. BPQDs were found to enhance the hole extraction at the anode side of p-i-n planar hybrid perovskite solar cells due to their high mobility and their well-matched band alignment with poly(3,4-ethylenedioxythiophene):poly(styrene sulfonate) (PEDOT: PSS) and methylammonium lead triiodide (MAPbI_3_) [122]. The PCE of the solar cells was improved with the addition of BPQDs from 14.10 to 16.69%. Furthermore, BPNS can improve the photostability of MAPbI_3_-based perovskite solar cells. After 1000 h of continuous illumination, MAPbI_3_/BPNS cells retained approximately 94% of their initial efficiency, while MAPbI_3_ cells without incorporated BPNS retained only 30% [123].

### 8.2. Sensors and Detectors Made of BPNS

BPNS are a promising material for detecting and sensing due to their thickness-dependent, tunable band gap, which covers a wide spectrum of light; high mobility, which enhances the sensitivity and speed of sensing devices; a reactive surface for functionalization and modification; sensitivity to mechanical stress; biocompatibility; and a high surface-to-volume ratio [10,100]. However, BPNS are sensitive to environmental factors and can degrade quickly; hence, strategies such as coatings, encapsulation, and functionalization are necessary to stabilize BPNS-based sensors. BPNS have been applied in many different sensing applications, including gas, bio, chemical, optical, strain, temperature, and humidity sensors [10,100,115]. Due to length constraints, this review will highlight gas and biosensors.

BPNS’s high surface-to-volume ratio, excellent optical and electric properties, and sensitivity to surface adsorption make it well-suited for gas sensors. Exfoliated BPNS dispersed in acetone were dropped onto interdigital Ag electrodes to detect ammonia (NH_3_) [112]. The sensor operated well at room temperature, exhibiting high sensitivity, selectivity, and repeatability, with a low limit of detection (LOD) of 100 ppb. Depending on the sonication duration during exfoliation, the BPNS-based sensor exhibited different responses to NH_3_. The largest response was to 100 ppm NH_3_ at 121% after 60 min of sonication [112]. While BPNS in Han et al.’s study was found to have a higher response to NH_3_, typically, BPNS has a better response and higher binding energy to NO_x_. In a comparison of the adsorption of carbon monoxide (CO), CO_2_, NH_3_, nitric oxide (NO), and nitrogen dioxide (NO_2_) gas molecules, BPNS is most sensitive to NO and NO_2_ [113]. When gas molecules adsorb onto monolayer BPNS, charge transfer is induced, and the electric current can either be reduced or increased, changing the resistance, which can be measured experimentally. However, BPNS easily degrades when exposed to strongly oxidizing NO_2_ gas. Incorporation of benzyl viologen (BV) was found to improve recovery characteristics, repeatability, humidity-repelling features, and selectivity [124]. Furthermore, BPNS/BV sensors and BNPS sensors have almost the same response to 50 ppb NO_2_ gas with a change in electrical resistance of −33% and −32%, respectively. This indicates that the incorporation of BV was able to improve sensor recovery, repeatability, and environmental stability without compromising its sensitivity to NO_2_.

The biocompatibility and the ability to be functionalized with targeting biomolecules allow BPNS to be used in biosensors. BPNS-based FET biosensors have attracted growing interest due to their excellent performance. For example, a BPNS-based FET biosensor was constructed using exfoliated BPNS as the sensing channel and aluminum oxide (Al_2_O_3_) thin film as the dielectric layer [125]. Gold nanoparticles labeled with anti-human immunoglobulin G (IgG) antibodies were sputtered onto the surface. When the antigen, human IgG, was bound to the antibody, the electrical conductivity of BPNS was altered and recorded as the sensing signal. The sensor was stable in ambient conditions for one week and had a LOD of 10 ng mL^−1^ [125]. In surface plasmon resonance (SPR) biosensors, sensitivity is commonly expressed in degrees per refractive index unit (° RIU^−1^), where RIU denotes a refractive index unit and the value represents the change in resonance angle for a unit change in the refractive index of the sensing medium. The Si-BP SPR biosensor exhibited a sensitivity of 184.6° RIU^−1^, meaning a 184.6° shift in the resonance angle per refractive index unit, whereas incorporation of a WS_2_ protective layer reduced the sensitivity to 163.1° RIU^−1^ while improving the environmental stability of BP [117]. The performance of BPNS-based SPR biosensors is comparable to that of SPR biosensors made of Si and other 2D materials. Another example of a biosensor is a fiber-optic biosensor made by integrating BPNS with a largely tilted fiber grating that uses anti-human neuron-specific enolase (anti-NSE) as the sensing element [126]. The anti-NSE would bind NSE, a cancer biomarker, causing changes in the local refractive index, which can be detected as a real-time optical signal. The fiber-optic sensor had a LOD of 1.0 pg mL^−1^, more sensitive than graphene oxide and gold nanoparticle-based biosensors [126].

### 8.3. BPNS Applied in the Field of Nanomedicine

The use of nanomaterials for medical applications is known as nanomedicine. It involves designing and fabricating nanostructured systems to act as therapeutic agents targeting specific sites, as photosensitizers in photothermal and photodynamic therapy, or as imaging agents and probes [127]. Cytotoxicity, biodegradation, immune interactions, and clearance are important considerations for the biomedical application of BPNS. In vitro studies have often reported relatively high cell viability within the concentration ranges examined. However, the biological response varies with particle dimensions, concentration, exposure time, surface chemistry, and cell type [16,127]. Surface modification can further alter this response by modulating colloidal stability, cellular uptake, and protein interactions in the biological environment. For example, protein-corona formation on BP nanomaterials has been shown to influence macrophage uptake and inflammatory signaling, indicating that apparently low cytotoxicity in conventional cell-viability assays does not necessarily imply complete biological inertness [16].

In vivo evidence is more limited and has largely been obtained using formulated or surface-modified BP systems rather than unmodified BPNS. Shao et al. demonstrated biodegradation and low short-term toxicity of PLGA-encapsulated BP quantum dots in a mouse tumor model, where the polymer coating also controlled the degradation rate of the BP component [15]. Thus, biodegradability and biocompatibility should be considered formulation- and condition-dependent rather than intrinsic properties of all BP nanomaterials. Degradation rates depend on factors including access to oxygen and water, pH, particle size, and protective surface coatings [15,127]. Although available studies support the biomedical potential of BP-based nanomaterials, additional long-term and dose-dependent in vivo studies are required to establish their systemic toxicity, immune effects, biodistribution, and clearance.

Sun et al. investigated PEGylated BPNS nanoparticles that could convert NIR light into heat and be used for photoacoustic imaging and photothermal therapy [25]. The PEGylated BPNS nanoparticles had excellent biocompatibility and photostability, and could accumulate and be retained in tumors, where irradiation would result in the killing of the tumor cells. In mice, treatment with PEGylated BPNS nanoparticles and laser irradiation resulted in shrinking and scabbing of the tumors after three days, and all mice survived for 42 days until they were sacrificed. In comparison, the control mice had a mean lifespan of 18–32 days [25]. In another study, BPNS were found to be able to cross the blood–brain barrier under NIR laser irradiation and capture Cu^2+^ ions in the brain, protecting from the progression of neurodegenerative disorders [128]. BPNS have many possibilities in nanomedicine, but more research is required on their cytotoxic effects and BPNS-based therapeutic systems.

## 9. Challenges of BPNS

One major challenge is maintaining the mechanical and thermal performance of BPNS under environmental cycling conditions. Repeated exposure to ambient air during heating and cooling cycles can accelerate oxidation and induce mechanical failure through layer delamination and crack propagation [71,104]. Encapsulation using materials like hexagonal boron nitride (h-BN) or atomic layer deposition (ALD) coatings has been shown to mitigate these effects. Most mechanical tests have been conducted on exfoliated flakes, which vary significantly in quality and thickness. For BPNS to be reliably integrated into scalable technologies, standardized methods for large-area mechanical testing and stress–strain profiling must be developed. These include in situ nanoindentation, bending tests, and strain mapping under real operating conditions [106]. As BPNS become integrated into nanoelectronics and thermal management systems, their interaction with other materials becomes critical. Research is ongoing into BPNS-based thermal interface materials (TIMs) and composites that can leverage their anisotropic heat transport while improving mechanical robustness and oxidation resistance [129]. Tailoring interface bonding and functionalization will be essential for future packaging technologies.

Beyond thermal and mechanical limitations, several broader challenges continue to hinder the practical implementation of BPNS. These include scalable and reproducible synthesis, precise control over layer thickness and lateral dimensions, long-term environmental stability, batch-to-batch reproducibility, and reliable large-scale surface functionalization. Furthermore, additional studies are needed to improve device integration, establish standardized characterization protocols, and evaluate the long-term biocompatibility, biodegradation, and biosafety of BPNS-based nanomaterials for biomedical applications. Addressing these challenges will accelerate the translation of BPNS from laboratory research to commercial electronic, sensing, energy, and biomedical technologies.

## 10. Conclusions

In conclusion, BPNS stand out as a versatile 2D material with unique optical and electronic properties that are highly favorable for a wide array of applications in nanotechnology. Its tunable direct band gap, which varies significantly with layer thickness, along with its anisotropic optical responses, positions BPNS as a key player in advancing photonic and electronic devices. The ability to manipulate its nonlinear optical properties opens new avenues for the development of advanced laser technologies and ultrafast optics.

The promising applications of BPNS in solar cells demonstrate their potential to enhance power conversion efficiencies through innovative heterostructure designs. Furthermore, its remarkable sensitivity in sensor technologies underscores the capability of BPNS to revolutionize detection methods across various domains, from environmental monitoring to biomedical diagnostics. In the realm of nanomedicine, biocompatibility and biodegradability of BPNS highlight their suitability for therapeutic applications, particularly in photothermal therapy and drug delivery. However, the successful translation of BPNS from the laboratory to real-world applications is contingent upon overcoming significant challenges. These include developing scalable synthesis methods that maintain the material’s quality and stability, as well as addressing environmental degradation issues.

Future research should prioritize the properties of BPNS exploration in practical contexts, including the development of robust protective coatings and stabilization strategies. Additionally, investigations into hybrid materials that combine BPNS with other 2D materials could yield enhanced functionalities and broaden their applicability. By leveraging the unique characteristics of BPNS, we can pave the way for groundbreaking advancements in nanotechnology, electronics, and biomedicine, ultimately driving innovation across multiple fields.

## Figures and Tables

**Table 1:** Comparison of dimensions, scale, and limitations of BP synthesis methods.

Method	Thickness & Lateral Dimensions	Yield/Scale	Defects/ Impurities	Oxidation Stability
Mechanical [1,13]	Monolayer to few-layer; µm-scale flakes	Low areal/mass throughput	Low lattice damage; adhesive/transfer residue possible	Rapid photooxidation if exposed
Liquid-phase [21,22,23]	Few- to multilayer; size-selected µm flakes	Batch dispersions; ~0.1–0.4 mg mL^−1^ reported [21]	Sonication/edge defects; solvent residue	Solvent or wrapping delays oxidation
BPQDs by sonication [24]	~1.5 nm thickness; ~2.6 nm laterally	Colloidal batches, not continuous films	High density of reactive edges	PEG/passivation can improve stability [24,25]
Electrochemical [26,37,41]	Predominantly 3–5 layers; ~0.83 µm mean lateral size [41]	Wafer-scale assembled networks [34], not one flake	Intercalant residues; junctions between flakes	Exposed sheets oxidize, protected transfer needed
Ball milling [28]	Variable particle thickness and lateral size	Powder-batch processing; no standard yield	Lattice defects; milling-media residues	Fresh surfaces air-sensitive
Sn/SnI_4_ vapor transport [29,32]	Bulk BP plates several mm; further exfoliation needed	Sealed-ampoule crystal batches	Potential Sn/I or mineralizer-phase residues	Bulk feedstock more protected; exfoliated surfaces degrade
Seeded vapor growth [19]	Few–hundreds nm thick; up to mm laterally	Direct millimeter-scale films demonstrated	Au_3_SnP_7_ seed phases; thickness uniformity	Surface oxidation requires mitigation
High-pressure conversion [28,33]	Bulk/polycrystalline BP; size depends on apparatus	High-pressure batch, not direct monolayer yield	Unconverted phases; cell-material contamination	Exfoliated product remains air-sensitive
Pulsed laser deposition + high pressure [27,34]	~40 nm/4 mm (thermal) [27]; ~18 nm/cm-scale (PLD) [34]	Demonstrated mm–cm films; wafer scale proposed	Nanocrystallinity [27]; pressure/annealing constraints	Film protection remains necessary

Abbreviations are as follows: black phosphorus (BP), black phosphorus quantum dot (BPQD), gold-tin-polyphosphate (Au_3_SnP_7_), iodine (I), polyethylene glycol (PEG), tin (Sn), and tin(IV) iodide (SnI_4_).

## Data Availability

No new data were created or analyzed in this study. Data sharing does not apply to this article.
